# Supplementary material for: Clinical practices underlie COVID-19 patient respiratory microbiome composition and its interactions with the host
Source: Nat Commun. 2021 Oct 29;12:6243. doi: 10.1038/s41467-021-26500-8 (PMC8556379; doi:10.1038/s41467-021-26500-8)
Supplement: Supplementary file 1 — Supplementary Information [file 41467_2021_26500_MOESM1_ESM.pdf]

Supplementary Information for:

Clinical practices underlie COVID-19 patient respiratory microbiome composition and its interactions with the host

Verónica Lloréns-Rico[1,2], Ann C. Gregory[1,2], Johan Van Weyenbergh[3], Sander Jansen[4], Tina Van Buyten[4], Junbin Qian[5,6], Marcos Braz[3], Soraya Maria Menezes[3], Pierre Van Mol[7,8,9], Lore Vanderbeke[10], Christophe Dooms[9,11], Jan Gunst[12], Greet Hermans[12], Philippe Meersseman[13], CONTAGIOUS collaborators, Els Wauters[9,11], Johan Neyts[4], Diether Lambrechts[7,8], Joost Wauters[13,14], Jeroen Raes[1,2,14,15]

[1] Laboratory of Molecular Bacteriology, Department of Microbiology and Immunology, Rega Institute, KU Leuven, Belgium
[2] Center for Microbiology, VIB, Leuven, Belgium
[3] Laboratory for Clinical and Evolutionary Virology, Department of Microbiology and Immunology, Rega Institute, KU Leuven, Belgium
[4] Laboratory of Virology and Chemotherapy, Department of Microbiology, Immunology and Transplantation, Rega Institute, KU Leuven, Belgium
[5] Zhejiang Provincial Key Laboratory of Precision Diagnosis and Therapy for Major Gynecological Diseases, Women's Hospital, Zhejiang University School of Medicine, Hangzhou, China
[6] Institute of Genetics, Zhejiang University School of Medicine, Hangzhou, China
[7] Laboratory of Translational Genetics, Department of Human Genetics, KU Leuven, Belgium
[8] VIB Center for Cancer Biology, VIB, Leuven, Belgium
[9] Department of Pneumology, University Hospitals Leuven, Belgium
[10] Laboratory of Clinical Bacteriology and Mycology, Department of Microbiology, Immunology and Transplantation, KU Leuven, Belgium
[11] Laboratory of Respiratory Diseases and Thoracic Surgery (BREATHE), Department of Chronic Diseases and Metabolism, KU Leuven, Belgium
[12] Laboratory of Intensive Care Medicine, Department of Cellular and Molecular Medicine, KU Leuven, Belgium
[13] Laboratory for Clinical Infectious and Inflammatory Disorders, Department of Microbiology, Immunology and Transplantation, KU Leuven, Belgium
[14] These authors contributed equally
[15] Corresponding author: jeroen.raes@kuleuven.vib.be

This file includes:

Supplementary Figures 1-5

Supplementary Tables 1-2

Supplementary Figure 1

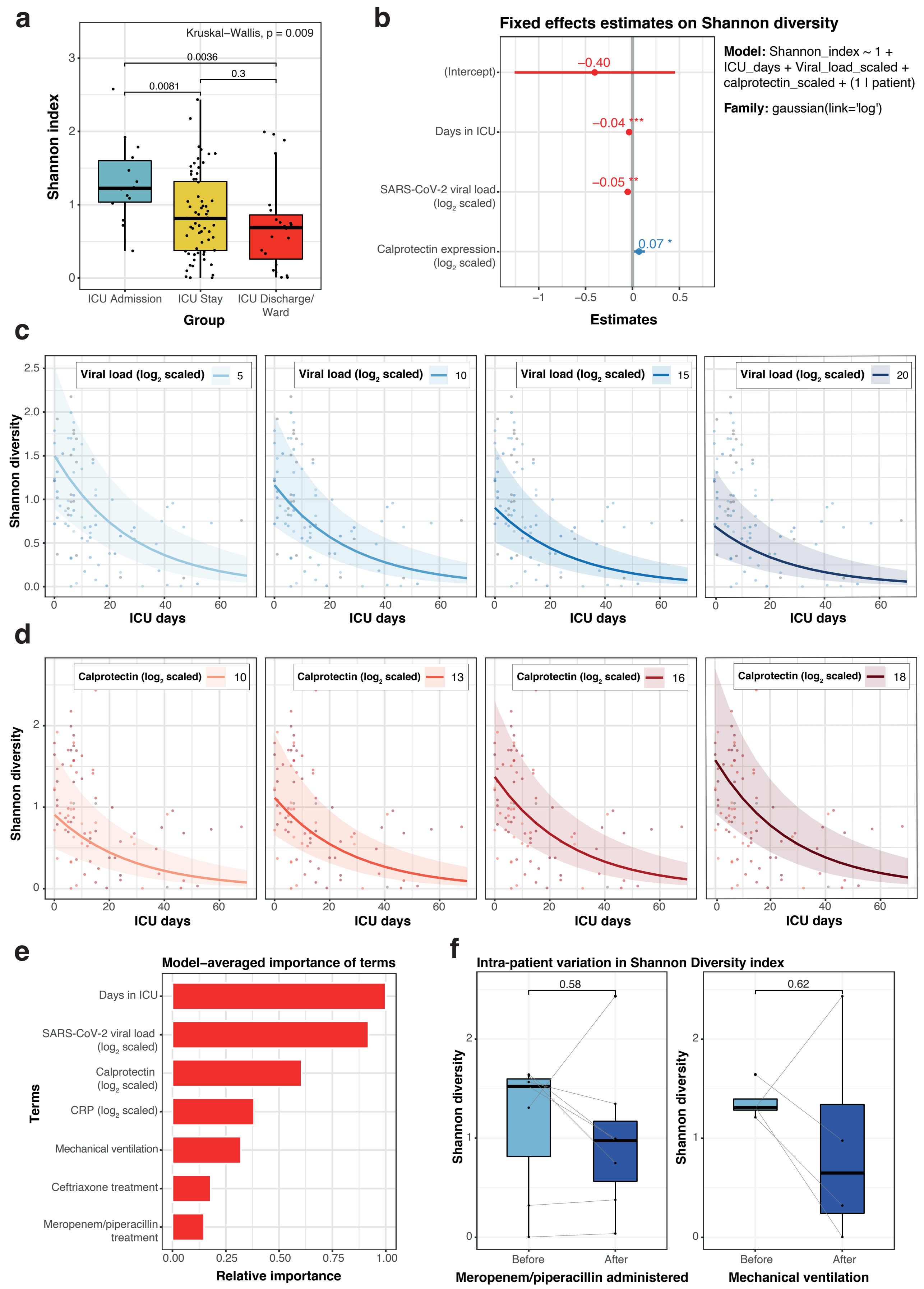

Supplementary Figure 1. Alpha diversity in the upper respiratory tract. **a.** Shannon diversity index of all samples (*n*=101), stratified by sampling moment: admission, throughout ICU stay or at ICU discharge/in ward. The *p*-value of a Kruskal-Wallis test, as well as those of pairwise post-hoc Dunn tests (two-sided), are shown. **b.** Forest plot of the fixed effects estimates of the variables selected in the best model predicting Shannon diversity index (based on *n*=101 samples). Selected fixed effects are length of ICU stay (two-sided Wald test, *p*-value = $1.41 \cdot 10^{-6}$), SARS-CoV-2 viral load (two-sided Wald test, *p*-value = 0.001) and calprotectin (S100A8) mRNA levels (two-sided Wald test, *p*-value = 0.02). The center points and the values above indicate the fixed effect estimates of the variables selected in the best model, while the horizontal lines span their 95% confidence intervals. **c.** Effect of the length of ICU stay and SARS-CoV-2 viral load on upper respiratory tract microbiome diversity. Each plot shows, for a different level of SARS-CoV-2 viral load (selected within the range of observed data), the model-predicted Shannon index as a function of the days in ICU (dark line). Shaded areas surrounding the line correspond to the 95% confidence intervals. **d.** Same as (c) but showing the association of the length of ICU stay and calprotectin gene expression levels with upper respiratory tract microbiome diversity. **e.** Model-averaged relative importance of each of the variables selected (only for fixed effects). **f.** Intra-patient differences of alpha-diversity (two-sided Wilcoxon signed-rank tests), before and after administration of meropenem/piperacillin-tazobactam (left, *n*=14 samples from 7 patients) or mechanical ventilation (right, *n*=8 samples from 4 patients). For (a,f), boxplots span from the first until the third quartile of the data distribution, and the horizontal line indicates the median value of the data. The whiskers extend from the quartiles until the last data point within 1.5 times the interquartile range, with outliers beyond. Individual data points are also represented. Asterisks denote significance as follows: * = *p*-value ≤ 0.05; ** = *p*-value ≤ 0.01; *** = *p*-value ≤ 0.001; **** = *p*-value ≤ 0.0001. Source data are provided as a Source Data file.

Supplementary Figure 2

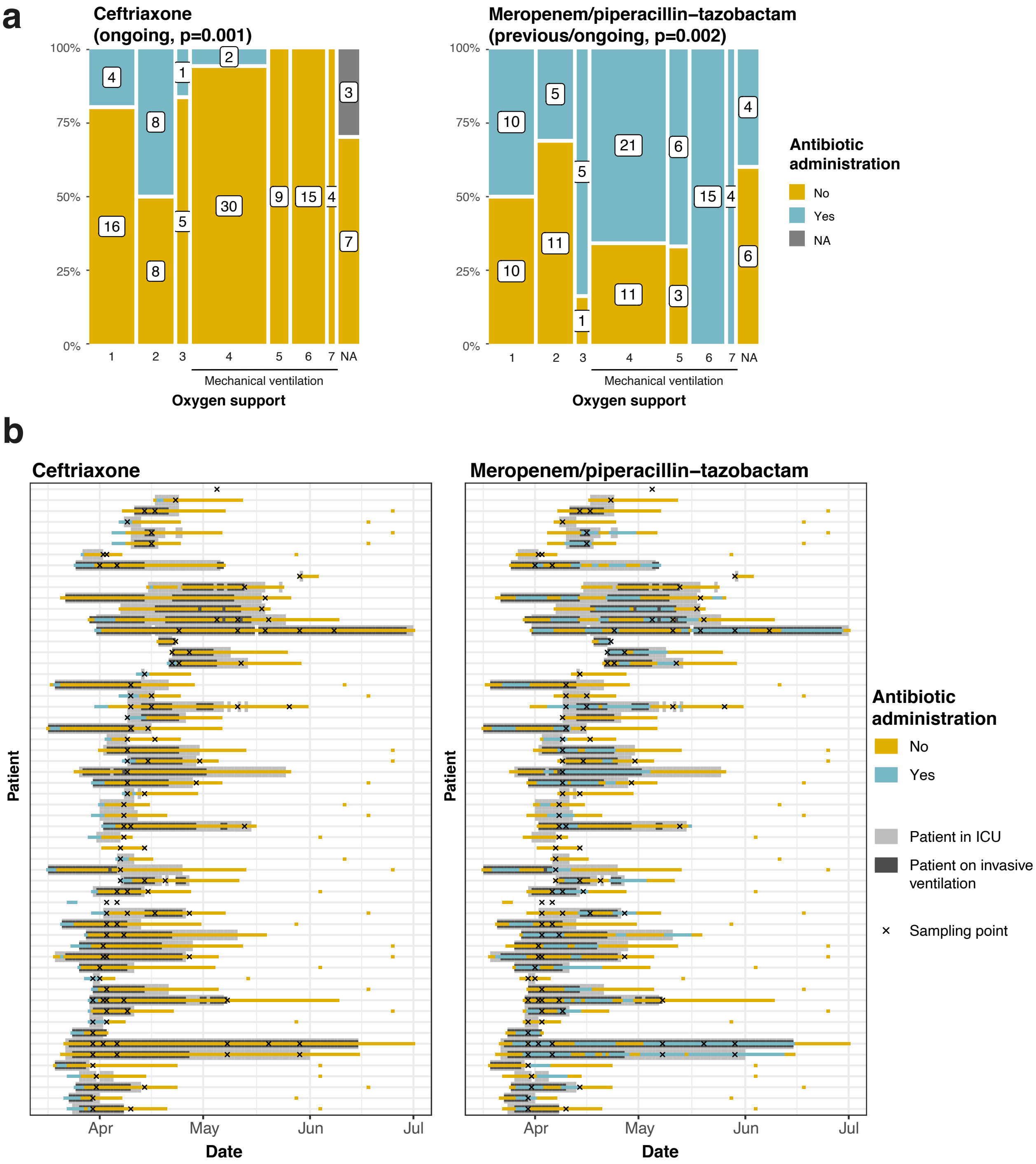

Supplementary Figure 2. Association of antibiotics and mechanical ventilation. **a.** Mosaic plots showing, for each category of oxygen support, the proportion of samples receiving ceftriaxone administration (current administration on day of sampling, left) or the proportion of samples having received meropenem or piperacillin-tazobactam (ongoing or previous treatment, right). *P*-values denote the significance of two-tailed Chi-squared tests for these associations. The different oxygen support levels are: 1-oxygen flow (via nasal

cannula); 2-high flow oxygen support; 3-non-invasive ventilation (CPAP, BIPAP); 4-invasive ventilation; 5-prone ventilation; 6-extra corporeal membrane oxygenation (ECMO); 7-nitric oxide inhalation. Levels 4-7 correspond to mechanically ventilated patients. **b.** Longitudinal sampling of patients (*n*=58 patients), showing specific antibiotic administration. Each line represents one patient. Yellow lines represent no-specific antibiotic administration for the spanned period; blue lines represent antibiotic was administered during that period. Shaded areas in light gray represent ICU stay, whilst areas in dark gray represent periods with the patient receiving mechanical ventilation. Crosses indicate the timepoints where swab samples were obtained for microbiome analyses. Individual yellow points at later times represent follow-up visits. Source data are provided as a Source Data file.

Supplementary Figure 3

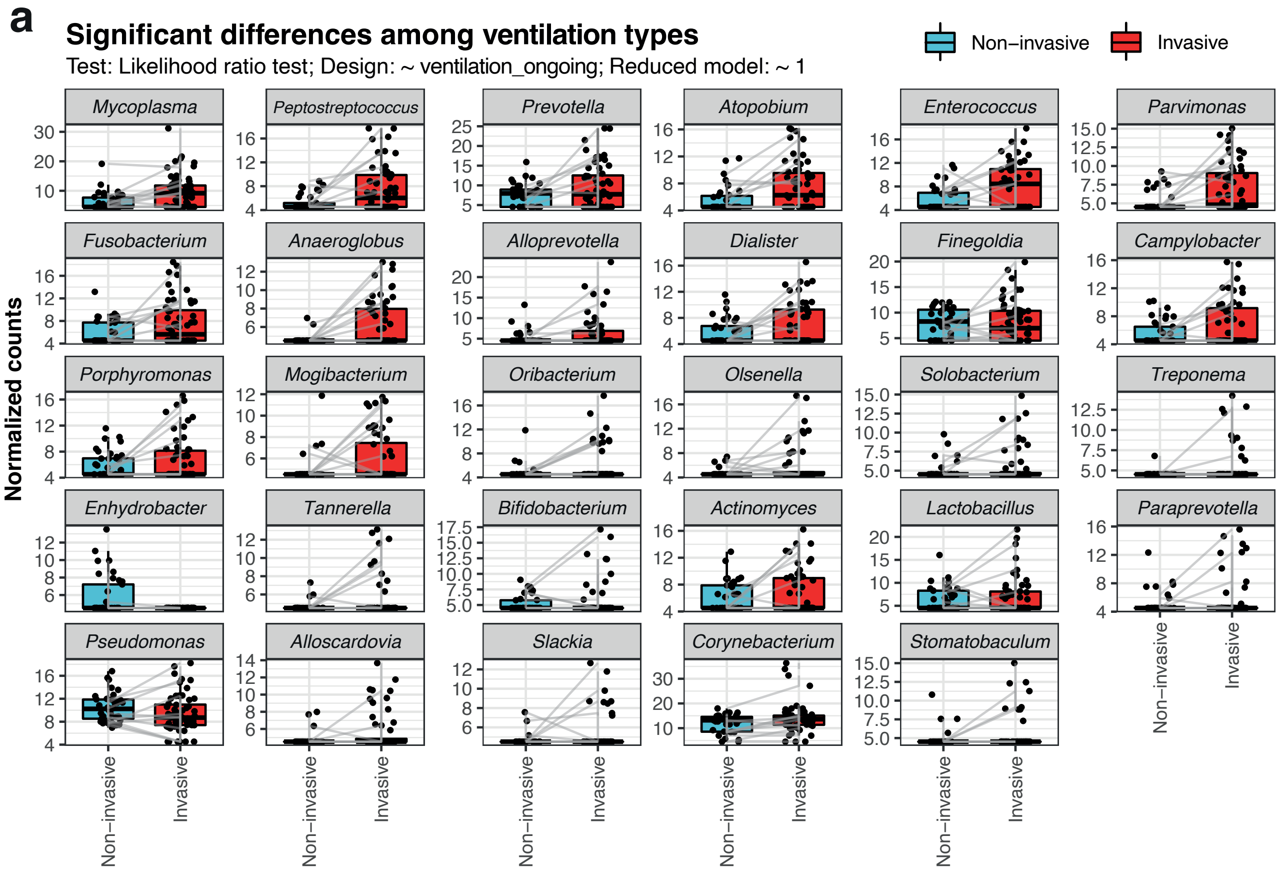

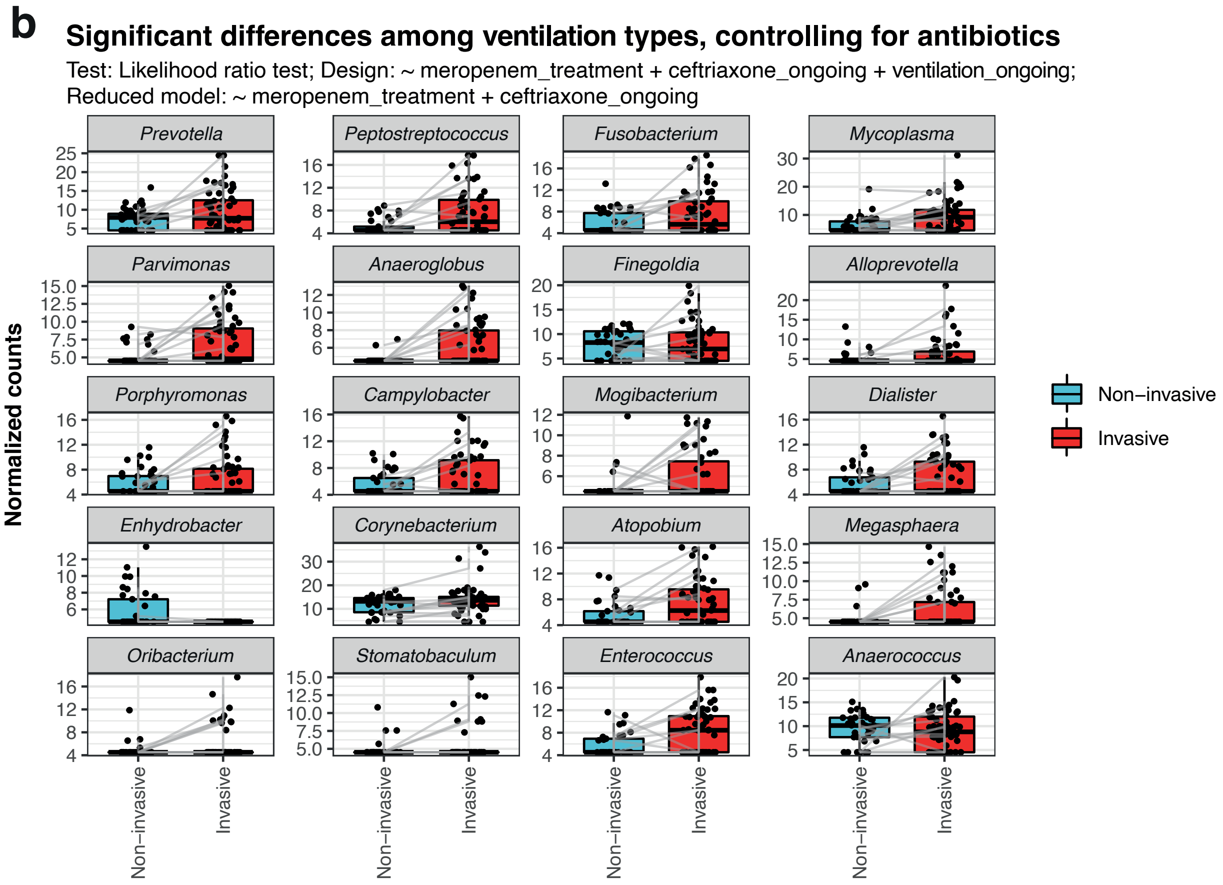

Supplementary Figure 3. Differentially abundant taxa between oxygen support types (*n*=101 samples). **a.** The 29 taxa whose abundance is significantly different between non-invasive and invasive ventilation are represented. **b.** The 20 taxa whose abundance is significantly different between ventilation types, after controlling for antibiotic usage, are represented. Boxplots span from the first until the third quartile of the data distribution, and the horizontal line indicates the median value of the data. The whiskers extend from the quartiles until the last data point within 1.5 times the interquartile range, with outliers beyond. Individual data points are also represented. Gray lines join samples pertaining to the same patient, taken at different time points. Source data are provided as a Source Data file.

Supplementary Figure 4

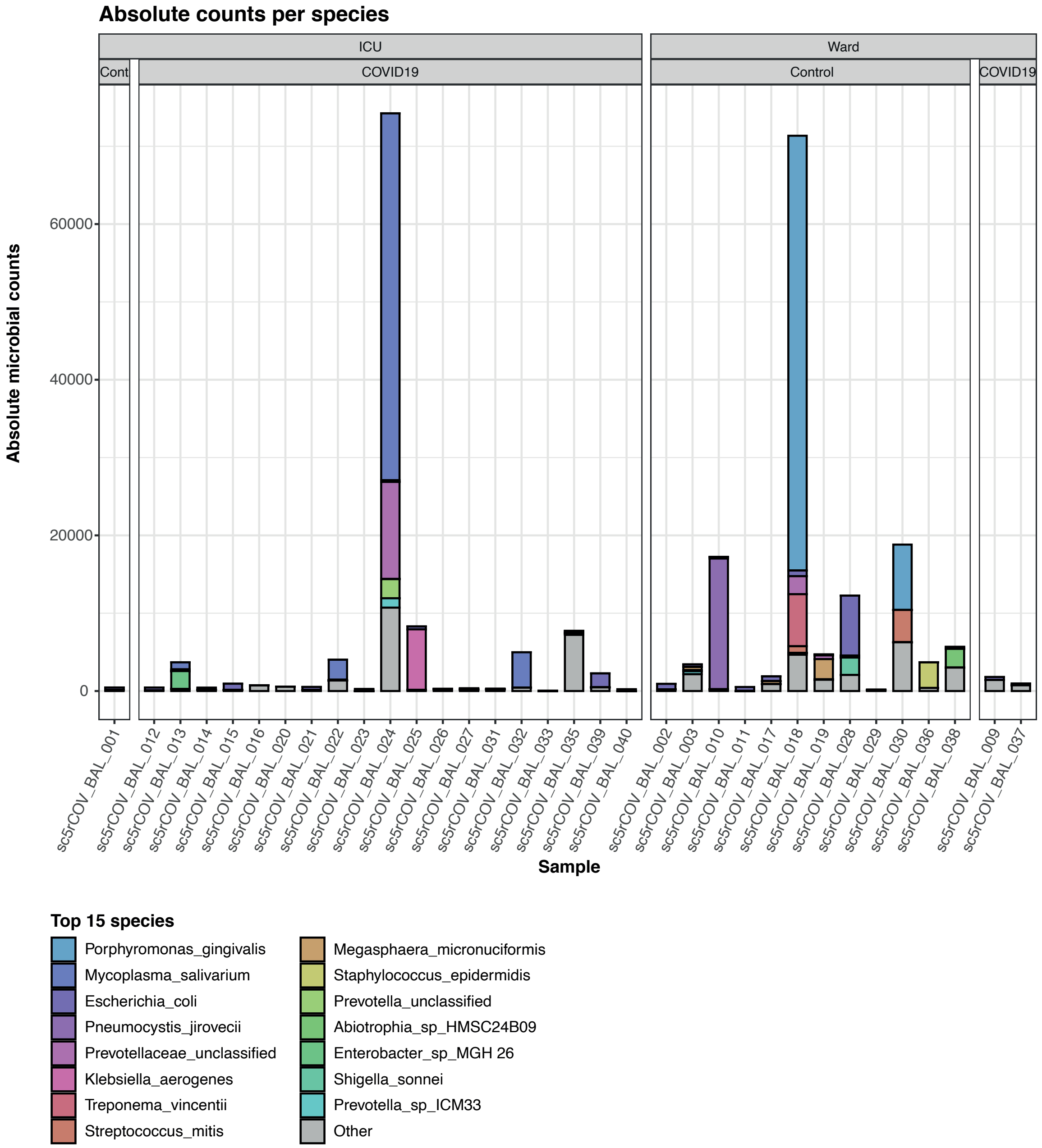

Supplementary Figure 4. Absolute microbial read counts in single-cell RNA-seq data from BAL samples. The top 15 species detected in our analyses are depicted. Samples are grouped by disease type (control for non-COVID-19 pneumonia patients, or COVID-19) and hospital stay (ICU or ward). Source data are provided as a Source Data file.

Supplementary Figure 5

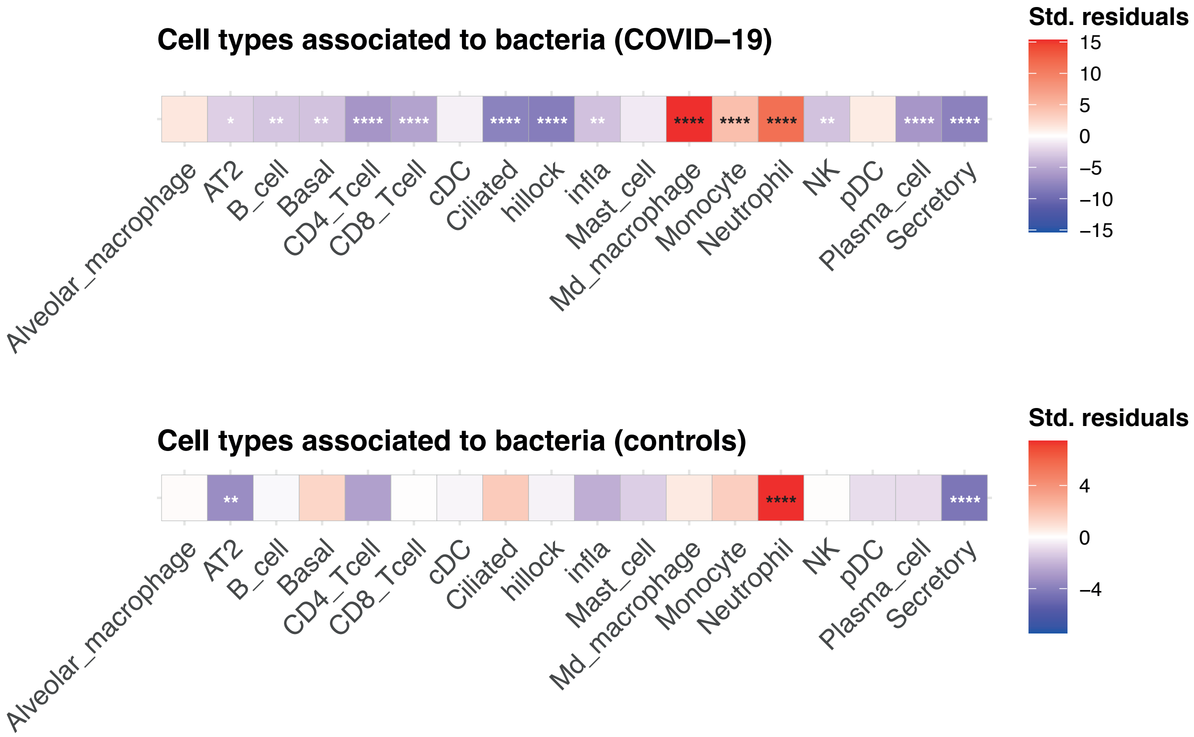

Supplementary Figure 5. Associations of specific cell types with bacteria, for COVID-19 and control samples. The colors represent the strength of the association as the standardized residuals of a two-tailed Chi-squared test. Red colors indicate a positive association (i.e. enrichment) of bacteria for each cell type. Blue colors indicate a negative association (i.e. depletion) of bacteria for a given cell type. Asterisks denote significance after multiple testing correction (Benjamini-Hochberg method) as follows: * = *p*-value ≤ 0.05; ** = *p*-value ≤ 0.01; *** = *p*-value ≤ 0.001; **** = *p*-value ≤ 0.0001. Exact *p*-values can be found in Supplementary Data 3. Source data are provided as a Source Data file.

Supplementary Table 1: Alpha diversity of the upper respiratory tract microbiome

Sample ID	Shannon Diversity Index	Sample ID	Shannon Diversity Index
COVID_001	1.507331529	COVID_052	0.002956549
COVID_002	0.076276906	COVID_053	0.018112562
COVID_003	0.632528006	COVID_054	0.038482362
COVID_004	2.17645435	COVID_055	0.872418708
COVID_005	0.008729568	COVID_056	1.791131332
COVID_006	0.796849454	COVID_057	1.70064693
COVID_007	1.425760729	COVID_058	0.545403021
COVID_008	0.758024383	COVID_059	0.554549522
COVID_009	0.681291882	COVID_060	1.414493641
COVID_010	1.045052242	COVID_061	1.21182799
COVID_011	1.693732089	COVID_062	0.321862569
COVID_012	0.09767734	COVID_063	0.378701653
COVID_013	0.180389239	COVID_064	1.04647796
COVID_014	0.251794687	COVID_065	0.562141195
COVID_015	0.810315428	COVID_066	0.687331013
COVID_016	0.676583152	COVID_067	0.676870851
COVID_017	1.052936063	COVID_068	0.577968368
COVID_018	1.92054897	COVID_069	1.235237521
COVID_019	1.961458273	COVID_070	1.314642189
COVID_020	0.821230207	COVID_071	0.007101196
COVID_021	0.715641808	COVID_072	0.003734165
COVID_022	0.369987984	COVID_073	1.125597421
COVID_023	0.695463015	COVID_074	0.377516747
COVID_024	0.749688484	COVID_075	1.468565118
COVID_025	1.630324764	COVID_076	0.996008082
COVID_026	0.720439889	COVID_077	1.523185746
COVID_027	0.867560683	COVID_078	1.786217189
COVID_028	0.718423252	COVID_079	0.894916982
COVID_029	0.924729885	COVID_080	0.983223645
COVID_030	1.110617561	COVID_081	0.745665044
COVID_031	0.182127117	COVID_082	0.340979719
COVID_032	1.456399162	COVID_083	0.813343765
COVID_033	1.752899535	COVID_084	0.318806792
COVID_034	0.330882911	COVID_085	0.162934327
COVID_035	0.173539357	COVID_086	0.031156754
COVID_036	0.246134002	COVID_087	0.955586383
COVID_037	0.615873174	COVID_088	0.486722795
COVID_038	1.01902735	COVID_089	0.912312951
COVID_039	1.308757877	COVID_090	0.727692874
COVID_040	0.105254179	COVID_091	0.634717491
COVID_041	2.434646886	COVID_092	2.578902469
COVID_042	1.567928633	COVID_093	1.575187127
COVID_043	0.706377755	COVID_094	0.521516603
COVID_044	1.34794981	COVID_095	1.698857525
COVID_045	1.643872121	COVID_096	1.993031932
COVID_046	0.977358713	COVID_097	0.972059987
COVID_047	0.333734377	COVID_098	0.816906462
COVID_048	1.347014623	COVID_099	1.088010274
COVID_049	0.788129672	COVID_100	0.36632322
COVID_050	0.356666066	COVID_101	0.400595893
COVID_051	1.881373172		

Supplementary Table 2: Contingency table showing host cells with identified bacterial or viral reads

	**SARS-CoV-2 negative cells**	**SARS-CoV-2 positive cells**
**No bacteria detected**	31868	342
**Bacteria-associated host cells**	1032	1
