# Supplementary material for: Clinical practices underlie COVID-19 patient respiratory microbiome composition and its interactions with the host
Source: Nat Commun. 2021 Oct 29;12:6243. doi: 10.1038/s41467-021-26500-8 (PMC8556379; doi:10.1038/s41467-021-26500-8)
Supplement: Supplementary file 2 — Description of Additional Supplementary Files [file 41467_2021_26500_MOESM2_ESM.docx]

**Description of Additional Supplementary Files**

**File Name:** Supplementary Data 1

**Description:** Covariates affecting respiratory microbiome composition, resulting from the dbRDA analysis. Each row shows one covariate tested, with the explanation of the covariate and the results of the dbRDA analyses, showing the *F*-statistic, the (adjusted) *R* squared, and the (Benjamini-Hochberg corrected) *p*-value.

**File Name:** Supplementary Data 2

**Description:** Differentially abundant taxa in the upper respiratory tract cohort. The table shows each of the taxa for which differential abundance was tested using DESeq2. The results from DESeq2’s likelihood ratio test (LRT) are shown, indicating which variables were included in the full and in the reduced model for each comparison.

**File Name:** Supplementary Data 3

**Description:** Associations of host cells with bacteria. The table indicates the associations of each host cell type with bacteria (any bacteria or specific genera) in COVID-19 patients or controls. The table shows the Chi-squared test *p*-values corresponding to these associations as well as the standardized residuals, and the manuscript Figure associated to each of the results shown.

**File Name:** Supplementary Data 4

**Description:** Host genes with higher expression in bacteria-associated cells. For each of the host cell subtypes enriched in bacterial associations, the table shows the results of the FindMarkers function in Seurat, to determine which genes are differentially expressed in the cells containing bacteria, compared to the cells where bacteria were not detected. The table shows, for each gene, the *p*-value of a two-sided Wilcoxon rank sum test, the average fold change, the fraction of cells with and without bacteria expressing the gene, and the Bonferroni-adjusted *p*-value.
