# Supplementary material for: Clinical practices underlie COVID-19 patient respiratory microbiome composition and its interactions with the host
Source: Nat Commun. 2021 Oct 29;12:6243. doi: 10.1038/s41467-021-26500-8 (PMC8556379; doi:10.1038/s41467-021-26500-8)
Supplement: Supplementary file 7 — Reporting Summary [file 41467_2021_26500_MOESM7_ESM.pdf]

nature portfolio

Corresponding author(s): Jeroen Raes

Last updated by author(s): Sep 23, 2021

Reporting Summary

Nature Portfolio wishes to improve the reproducibility of the work that we publish. This form provides structure for consistency and transparency in reporting. For further information on Nature Portfolio policies, see our Editorial Policies and the Editorial Policy Checklist.

Statistics

For all statistical analyses, confirm that the following items are present in the figure legend, table legend, main text, or Methods section.

n/a	Confirmed	
[ ]	[x]	The exact sample size (n) for each experimental group/condition, given as a discrete number and unit of measurement
[ ]	[x]	A statement on whether measurements were taken from distinct samples or whether the same sample was measured repeatedly
[ ]	[x]	The statistical test(s) used AND whether they are one- or two-sided<br>*Only common tests should be described solely by name; describe more complex techniques in the Methods section.*
[ ]	[x]	A description of all covariates tested
[ ]	[x]	A description of any assumptions or corrections, such as tests of normality and adjustment for multiple comparisons
[ ]	[x]	A full description of the statistical parameters including central tendency (e.g. means) or other basic estimates (e.g. regression coefficient) AND variation (e.g. standard deviation) or associated estimates of uncertainty (e.g. confidence intervals)
[ ]	[x]	For null hypothesis testing, the test statistic (e.g. F, t, r) with confidence intervals, effect sizes, degrees of freedom and P value noted<br>*Give P values as exact values whenever suitable.*
[x]	[ ]	For Bayesian analysis, information on the choice of priors and Markov chain Monte Carlo settings
[x]	[ ]	For hierarchical and complex designs, identification of the appropriate level for tests and full reporting of outcomes
[ ]	[x]	Estimates of effect sizes (e.g. Cohen's d, Pearson's r), indicating how they were calculated

Our web collection on statistics for biologists contains articles on many of the points above.

Software and code

Policy information about availability of computer code

Data collection	No software was used for data collection

Data analysis

For the 16S data analyses: reads were demultiplexed with LotuS v1.565 and processed following the DADA2 microbiome pipeline using the R packages DADA2 (v1.18.0) and phyloseq (v1.34.0). Taxonomy was assigned using the Ribosomal Database Project (RDP) classifier (v16) implemented in DADA2. For decontamination, we used the prevalence-based contaminant identification method in the R package decontam (v1.12.0). For the statitstical analyses, the following R packages were used: vegan (v2.5.7), phyloseq (v1.34.0), CoDaSeq (v0.99.6), DESeq2 (v1.30.1), Biostrings (v2.58.0), rstatix (v0.7.0), DECIPHER (v2.18.1), glmulti (v1.0.8) and lme4 (v1.1.27).

Single-cell data was processed to identify microbial reads as follows: Only read 2, containing the information on the cDNA, was used. Trimmomatic (v0.38) was used to remove trim low quality bases and discard short reads. Additionally, Prinseq++(v1.2) was used to discard reads with low-complexity stretches. Reads from human and potential sequencing artifacts (phage phiX174) were mapped with STAR (v2.7.1) and discarded. The remaining reads were mapped against bacterial genomes using a 2-step approach: first, we scanned the reads using mash screen (v2.0) against a custom database of 11685 microbial reference genomes including bacteria, archaea, fungi and viruses. Genomes likely to be present in the analyzed sample (selected using a threshold of at least two shared hashes from mash screen) were selected and reads were pseudoaligned to this subset of genomes using kallisto (v0.44.0). Bacterial reads were assigned their specific barcodes and UMIs as follows: read IDs from the mapped microbial reads were retrieved from the kallisto pseudoalignment (*.bam) output using SAMtools (v1.9). These unique read IDs were used to retrieve the specific barcodes and UMIs using the raw read 1 fastq files, thus assigning each barcode and UMI univocally to a microbial species and function. After data preprocessing of the scRNA-seq data, statistical analyses of the lower respiratory microbiome tract were done in R using the following packages: Seurat (v4.0.4) and chisq.posthoc.test (v0.1.2).

Additional R packages used for plotting and data wrangling are listed in the Github repository where the scripts used are available: https://github.com/raeslab/covid19_respiratory_microbiome

For manuscripts utilizing custom algorithms or software that are central to the research but not yet described in published literature, software must be made available to editors and reviewers. We strongly encourage code deposition in a community repository (e.g. GitHub). See the Nature Portfolio guidelines for submitting code & software for further information.

Data

Policy information about availability of data

All manuscripts must include a data availability statement. This statement should provide the following information, where applicable:

- Accession codes, unique identifiers, or web links for publicly available datasets
- A description of any restrictions on data availability
- For clinical datasets or third party data, please ensure that the statement adheres to our policy

The raw amplicon sequencing data generated in this study, as well as patient metadata, have been deposited in the European Genome-phenome Archive (EGA) repository under accession code EGAS00001004951 (https://ega-archive.org/studies/EGAS00001004951). These data are available under restricted access to comply with current European personal data protection regulations. Access can be obtained for research purposes within specific approved projects granted by the corresponding Data Access Committee (https://ega-archive.org/dacs/EGAC00001001901).
The single cell RNA-seq data was first described in a separate publication and deposited also in EGA with accession number EGAS00001004717 (https://ega-archive.org/studies/EGAS00001004717).

Field-specific reporting

Please select the one below that is the best fit for your research. If you are not sure, read the appropriate sections before making your selection.

[x] Life sciences   [ ] Behavioural & social sciences   [ ] Ecological, evolutionary & environmental sciences

For a reference copy of the document with all sections, see nature.com/documents/nr-reporting-summary-flat.pdf

Life sciences study design

All studies must disclose on these points even when the disclosure is negative.

Sample size

No sample size calculations were predetermined before patient inclusion in the study. The obtained sample size was sufficient to detect changes in taxa abundances between samples from non-mechanically ventilated (N=37) and mechanically ventilated patients (N=56) of a minimum effect size of 1.53; based on the average standard deviation of microbial taxa in our cohort (considering $\alpha=0.05$, $\beta=0.2$).

Data exclusions

For the 16S amplicon sequencing data analyses of the upper respiratory microbiome cohort, samples with <10,000 reads assigned at the genus level were not used (11 samples excluded).

Replication

As this study is part of a prospective observational trial, no replication cohort has been made available for replication studies.
Technical replicates were introduced of some samples as part of the quality control of the dataset. Once that technical reproducibility was assessed (by determining that Aitchison distances between replicates are significantly smaller than between different samples), technical replicates were pooled together and treated as a single, biological sample.

Randomization

Not applicable. Patients in the upper respiratory microbiome cohort were all diagnosed with COVID-19; patients in the lower respiratory cohort were classified according to their diagnosis: COVID-19 or non-COVID-19 pneumonia.

Blinding

Not applicable. Investigators were not blinded during the data collection or analyses, as this was not a randomized study and patients

recruited to the study were already hospitalized with either COVID19 or non-COVID19-pneumonia, and entered the study as such.

Reporting for specific materials, systems and methods

We require information from authors about some types of materials, experimental systems and methods used in many studies. Here, indicate whether each material, system or method listed is relevant to your study. If you are not sure if a list item applies to your research, read the appropriate section before selecting a response.

Materials & experimental systems

n/a	Involved in the study
☒	☐ Antibodies
☒	☐ Eukaryotic cell lines
☒	☐ Palaeontology and archaeology
☒	☐ Animals and other organisms
☐	☒ Human research participants
☐	☒ Clinical data
☒	☐ Dual use research of concern

Methods

n/a	Involved in the study
☒	☐ ChIP-seq
☒	☐ Flow cytometry
☒	☐ MRI-based neuroimaging

Human research participants

Policy information about studies involving human research participants

Population characteristics

A description of the two cohorts analyzed in this manuscript is available in Table 1.
For the upper respiratory cohort (N=58), average age is 61 years (range = 37-83), average BMI is 29 (22-47), 13 patients are female (22%) and 12 are diabetic (21%).
In the lower respiratory tract cohort (N=35), the average age is 64 years (range = 45-85), average BMI is 26 (16-36), 12 patients are female (34%) and 6 are diabetic (17%).

Recruitment

Patients were recruited after admission to UZ Leuven hospital with the following inclusion criteria: patients >/= 18 years old and hospitalized with PCR-confirmed and/or CT-confirmed SARS-CoV-2 infection and COVID-19 disease, or non-COVID-19 pneumonia for the case of controls in the lower respiratory tract.
Exclusion criteria were: age < 18 years old, no informed consent provided, and patients on cyclosporine/tacrolimus/sirolimus/everolimus therapy.
All patients in the study provided informed consent to participate.
A possible bias in the recruitment of the lower respiratory tract cohort lies in the fact that non-COVID19 pneumonia patients were mostly in ward as their condition was clinical to moderate, while COVID19 patients were mostly in the ICU. This could not be avoided as the patient recruitment and collection occurred during the 1st coronavirus wave in Belgium, where ICU units were largely occupied by COVID19 patients. The main consequence of this is that it is not possible to discern whether the effects observed between COVID19 patients and non-COVID19 pneumonia controls are due to the disease itself or due to the disease severity or clinical practices associated to ICU stay, as both factors were highly correlated.

Ethics oversight

The protocol was reviewed and approved by the Ethics Commissie UZ Leuven. All patiens were able to provide informed consent to participate in the study.

Note that full information on the approval of the study protocol must also be provided in the manuscript.

Clinical data

Policy information about clinical studies

All manuscripts should comply with the ICMJEguidelines for publication of clinical research and a completedCONSORT checklist must be included with all submissions.

Clinical trial registration

NCT04327570

Study protocol

A description of the observational trial can be found in https://clinicaltrials.gov/ct2/show/record/NCT04327570?view=record. For access to the entire protocol, the lead clinical investigator, Prof. Dr. Joost Wauters (joost.wauters@uzleuven.com), should be contacted.

Data collection

Patients included in this study were recruited between March and June upon admission at UZ Leuven hospital. Source data was obtained from the participants medical records, and was transferred pseudonymized to the researchers of this study.
Nasopharyngeal swabs for upper respiratory microbiome profiling were procured longitudinally at different timepoints throughout patient hospitalization as depicted in Figure 1a of this manuscript. Bronchoalveolar lavages were performed only when clinically indicated.

Outcomes	The correlation of immune profiling in COVID-19 patients with their microbiome was established as one of the secondary endpoints of the NCT04327570 clinical trial. This was achieved by correlating different immune features (such as mRNA levels of inflammation markers such as calprotectin, or relative abundances of immune cells) with the overall microbiota composition via dbRDA tests, as well as by the analyses of host-bacterial interactions in the single-cell RNA-seq data from the lower respiratory tract cohort.<br>The correlation of clinical practices with the microbiome of hospitalized COVID19 patients was not amongst the planned study outcomes.
